# Phycobilisomes: modular light‐harvesting systems of cyanobacteria and red algae

**DOI:** 10.1111/tpj.71065

**Published:** 2026-08-04

**Authors:** Henning Kirst, María Agustina Domínguez‐Martín

**Affiliations:** ^1^ Department of Genetics, Faculty of Science University of Córdoba, Campus Universitario de Rabanales Córdoba 14014 Spain; ^2^ Maimónides Biomedical Institute (IMIBIC) 14004 Córdoba Spain; ^3^ Campus of International Excellence in Agrifood, ceiA3 University of Córdoba Córdoba Spain; ^4^ Department of Biochemistry and Molecular Biology, Faculty of Science University of Córdoba, Campus Universitario de Rabanales Córdoba 14014 Spain

**Keywords:** phycobilisome, cyanobacteria, light‐harvesting, structure, photosynthesis, non‐photochemical quenching, OCP

## Abstract

Phycobilisomes (PBSs) are supramolecular pigment–protein complexes that function as the principal light‐harvesting antennae in cyanobacteria, red algae (Rhodophyta), and glaucophytes, and to a lesser extent in cryptophytes. Because the intrinsic chlorophyll‐based antennae of the photosystems absorb poorly in the green region of the spectrum, PBSs employ specialized bilin chromophores to capture complementary wavelengths that chlorophyll cannot efficiently harvest. Thus, PBSs broaden the photosynthetic active spectrum of oxygenic phototrophs and thereby increase their photosynthetic capacity. Structural studies using cryogenic electron microscopy and advanced spectroscopic techniques have revealed increasingly detailed insights into excitation energy‐transfer pathways and the bilin microenvironments that support highly efficient energy funneling with minimal loss. These approaches have also uncovered previously unrecognized conformational dynamics, although the functional significance of these motions remains unknown. Together, these findings portray PBSs as both ancient and highly adaptable molecular machines, central to the ecological success of cyanobacteria and other phycobiliprotein‐containing phototrophs, and still yielding new surprises as analytical techniques continue to improve. This review integrates foundational and recent advances in the structural organization, functional mechanisms, and emerging applications of PBSs. We examine PBS architectures and their distribution across lineages, energy‐transfer and photoprotective processes, structural dynamics, evolutionary trajectories, and the expanding landscape of biotechnological uses. These insights establish a foundation for future efforts to link PBS structure with ecological function and biotechnological innovation.

## Significance Statement

Phycobilisomes (PBS) are ancient, modular light‐harvesting machines whose architecture, pigment diversity, and regulatory flexibility support the ecological success of cyanobacteria and red algae. Recent breakthroughs resolve diverse PBS architectures at near‐atomic detail, revealing how these complexes achieve efficient light‐energy capture, assemble into higher‐order arrays, and dissipate potentially toxic excess excitation energy as harmless heat. This structural clarity provides a powerful foundation for biotechnological innovations, ranging from light‐capture devices to high‐yield protein fusion systems.

## INTRODUCTION

The harvesting of photons is the first step in photosynthesis, the biological process that converts solar energy into chemical energy (Croce & van Amerongen, 2014). To achieve this, photosynthetic organisms have evolved specialized structures that efficiently capture incident light, creating an excited electronic state whose energy is rapidly funneled to a reaction center and converted into separated charges that drive chemistry (Mirkovic et al., 2017; Scholes et al., 2011). Among the most widespread of light‐harvesting structures, particularly in cyanobacteria, are the phycobilisomes (PBSs) (Adir et al., 2020; Arteni et al., 2009; Bryant et al., 1979; Gantt et al., 1979; Glazer et al., 1983; MacColl, 1998; Tandeau de Marsac, 2003).

Light‐harvesting complexes are assemblies of pigments and proteins that absorb photons across a broad spectral range, thereby greatly increasing the effective absorption cross‐section of the reaction centers. Their role is essential because light is intrinsically dilute: even on a sunny day, a chlorophyll molecule absorbs no more than one photon every 0.1 sec (Blankenship, 2002). Without light‐harvesting antennae, reaction centers would remain inactive for most of the time. Increasing the photon‐absorption cross‐section of the photosystems II and I (PSII and PSI) is therefore critical, especially under light conditions lower than bright sunlight. This becomes particularly evident in the deep ocean, where organisms must harvest every available photon because ambient light levels are extremely low, often comparable to those in polar winter twilight, <1 μmol photons m^−2^ sec^−1^, which is 3–4 orders of magnitude lower than the about 2000 μmol photons m^−2^ sec^−1^ in bright sunlight. Moreover, because light quantity and quality vary substantially across natural habitats, antenna systems function as modular units that can be tuned to environmental demands (Blankenship, 2001, 2002, 2010; Melis, 2009). As a result, light‐harvesting complexes display remarkable diversity in pigment composition, pigment organization, and overall size. At the same time, the capture and management of light energy is inherently risky. Rapid fluctuations in light intensity, occurring on timescales of seconds, can lead to overexcitation of the photosynthetic machinery, causing photodamage and driving the formation of reactive oxygen species (ROS) that can lead to cell death (Eberhard et al., 2008). Thus, photosynthetic organisms need to have mechanisms in place to safely dissipate excess energy when needed, and recent work increasingly reveals that the architecture of PBSs allows light harvesting to be rapidly shut down at the antenna itself, providing an efficient route for dissipating excess energy.

Given that light harvesting initiates one of the most fundamental biochemical processes on Earth, evolutionary success has depended on the development of architectures, compositions, and regulatory mechanisms that optimize this function. PBSs exemplify this evolutionary refinement: they are modular, highly tunable antennae whose architecture, size, pigment complement, rod‐core organization, and linker proteins (LPs) directly reflect the light environment and ecological niche of the organism. In many respects, PBSs represent an elegant case of evolutionary ‘optical engineering’.

Research on PBSs dates back to 1836, when their constituent pigments were first described as brilliantly blue, re‐fluorescent, photolabile, and water‐soluble compounds released by the cyanobacterium *Oscillatoria* sp. (Esenbeck, 1836; Tandeau de Marsac, 2003). The PBS as a supramolecular complex was identified by Gantt and Conti in the 1960s (Gantt & Conti, 1966a, 1966b). In the 1980s and 90s, Huber and colleagues solved the first high‐resolution structures of phycobiliproteins (PBPs) using X‐ray crystallography (Brejc et al., 1995; Duerring et al., 1990; Ficner et al., 1992; Schirmer et al., 1985, 1986, 1987), marking the earliest structural characterization of light‐harvesting components. Negatively stained PBS particles had been examined by transmission electron microscopy (EM) since the 1970s (Bryant et al., 1976; Ducret et al., 1996; Glazer et al., 1983; Yamanaka et al., 1978), leading to the classic model of a tricylindrical core surrounded by radiating rods. Despite their suitability for EM due to their large size, major breakthroughs in PBS structural biology have occurred only in recent years, driven by advances in cryogenic electron microscopy (cryo‐EM) instrumentation, computational methods, and sample‐preparation technologies (Table 1). These developments have enabled the determination of intact PBS structures, detailed comparisons across PBS types, visualization of PBS–PSII supercomplexes (You et al., 2023; Zhang et al., 2024), and, through cryo‐electron tomography (cryo‐ET), the *in situ* ultrastructure of PBSs within the native cellular context (Li et al., 2021; Rast et al., 2019).

**Table 1:** Examples of most recent studies on PBS using cryo‐EM or cryo‐ET

Organism	PBS type	Technique and resolution	Main structural contribution	References
*Anabaena* sp. PCC 7120	PSI‐CpcL‐PBS	Single‐particle cryo‐EM Overall resolution of PSI‐CpcL‐PBS at 2.98 Å and CpcL‐PBS at 2.93 Å resolution	Cryo‐EM reveals the complete architecture of the PSI‐CpcL‐PBS supercomplex, showing how a three‐layered CpcL‐type phycobilisome docks onto a PSI tetramer through the transmembrane helix of CpcL. The structures resolve the precise arrangement of linkers, PC monomers, bilins, and chlorophylls, providing the structural basis for supercomplex assembly and excitation energy transfer.	Mao et al. (2026)
*Gloeobacter violaceus* 7421	Bundle‐shaped	Single‐particle cryo‐EM Overall resolution 3.37 and 2.8 Å. Low versus moderate‐light PBS assemblies.	Reveals two unique linker proteins (Lrc^91^ and Lrc^81^) that play a key role in the PBS structure. Uncovering long rods that contain an ApcA2–ApcB3–ApcD layer and short rods lack this layer.	Ma et al. (2025)
*Synechococcus elongatus* PCC 7942	Bicylindrical core	Single‐particle cryo‐EM at overall resolution of 3 Å	First near‐atomic structure of a bicylindrical core PBS; shows how C‐terminal ApcE compensates for the missing top APC cylinder to attach rods via CpcG. Analysis of the bilin distribution showed that distance of excitation energy transfer from top peripheral rods to the terminal emitters is approximately 15% shorter compared to the PBS with tricylindrical cores.	Zheng et al. (2025)
*Gloeobacter violaceus* 7421	Bundle‐shaped	Single‐particle cryo‐EM, resolution ranging from 2.72 to 3.67 Å	>10 MDa PBS with diverging, mobile rod bundles from a pentacylindrical APC core; identifies two multidomain linkers (Glr1262 and Glr2806) that maintain bundle architecture. Additionally, it shows *in vitro* differential no‐photochemical quenching via two Orange Carotenoid Protein (OCP)3 types.	Burtseva et al. (2025)
*Synechocystis* sp. PCC 6803	CpcL‐PBS	Single‐particle cryo‐EM, resolution at 2.6 Å.	Structure of compact CpcL‐type PBS that transfers directly to PSI; localizes the CpcD domain of ferredoxin: NADP^+^ oxidoreductase at the distal end responsible for PBS attachment.	Zheng et al. (2023)
*Anthocerotibacter panamensis*	Paddle‐shaped	Single‐particle cryo‐EM, resolution at 2.9 Å.	Rod‐less, paddle‐shaped PBS with chains of PC hexamers (CpcN) and two core subdomains (ApcH), lacking ApcD/F; energy‐transfer calculations show inefficient chains versus rods, illuminating PBS evolution.	Jiang et al. (2023)
*Porphyridium purpureum*	Hemiellipsoidal	Single‐particle cryo‐EM of low‐light PBS versus medium‐light PBS at resolution of 2.8 and 3.04 Å.	Compares 14.7 MDa (LL) versus 13.6 MDa (ML) PBS; LL has more closely coupled chromophore pairs and red‐shifted emission, but similar fluorescence quantum yield, giving a structural basis for PBS‐level light acclimation.	Dodson et al. (2023)
*Synechocystis* sp. PCC 6803	Hemidiscoidal	Single‐particle cryo‐EM of PBS–OCP complex at resolution of 1.6–2.1 Å.	The study reveals high‐resolution structures of the OCP bound to cyanobacterial PBSs, showing precisely how OCP docks onto specific PBS sites to trigger non‐photochemical quenching. They also showed its inherent flexibility.	Sauer et al. (2024)
*Synechocystis* sp. PCC 6803	Hemidiscoidal	Single‐particle cryo‐EM of PBS and PBS in complex with OCP. Overall resolution from 2.7 to 3.5 Å.	6.2 MDa PBS in three rod‐arrangement conformations plus an OCP‐bound quenched state; identifies a new linker (ApcG) on membrane‐facing side and shows that only one conformer binds OCP, defining a structural basis for regulation and quenching.	Dominguez‐Martin et al. (2022)
*Thermosynechococcus vulcanus*	Hemidiscoidal pentacylindrical	Single‐particle cryo‐EM of core and rod at a resolution of 3.7 and 4.2 Å, respectively.	Define how the pentacylindrical core is formed, identify key interactions between linker proteins and the bilin chromophores. It also indicates pathways for unidirectional energy transfer.	Kawakami et al. (2022)
*Anabaena* sp. PCC 7120 and *Synechococcus* sp. PCC 7002	Hemidiscoidal with pentacylindrical and tricylindrical	Single‐particle cryo‐EM at a resolution of 3.5 Å (7002) and 3.9 Å (7120).	Hemidiscoidal PBSs with common triangular cores but different extra hexamers; maps chromophore organization and shows aromatic residues in ApcD/F and linker proteins are critical for excitation energy transfer.	Zheng et al. (2021)
*Porphyridium purpureum* UTEX 2757	Hemiellipsoidal PBS–PSII supercomplex *in situ*	Cryo‐ET	In‐cell tomographic structure of hemiellipsoidal PBS–PSII arrays; each PBS contacts six PSII monomers via several connector proteins; identifies ApcD and α‐subunits as multiple energy‐delivery routes to PSII.	Li et al. (2021)
*Porphyridium purpureum*	PBS–PSII–PSI–LHC megacomplexes	Cryo‐ET + *in situ* single‐particle analysis	Near‐atomic tomographic structure of PBS–PSII–PSI–LHC megacomplex; details pigment network and interfaces, giving a structural framework for energy distribution between PSII and PSI in red algae.	You et al. (2023)
*Arthrospira* sp. FACHB439	PBS–PSII complex	Cryo‐ET with subtomogram averaging: STAgSPA with a 3.5 Å resolution.	The structure uncovers how neighboring PBSs and PSII dimers interact, highlighting a cooperative energy‐transfer process driven by multiple super‐PBS assemblies in cyanobacteria	Zhang et al. (2024)
*Porphyridium purpureum*	Hemiellipsoidal PBS	Single‐particle cryo‐EM at 2.82 Å resolution.	Higher resolution of a 14.7 MDa PBS. The structure shows how linker proteins shape the chromophores' local environment and indicates that aromatic residues within these linkers interact with the pigments in ways that fine‐tune their energy levels, enabling efficient, directional energy transfer.	Ma et al. (2020)
*Griffithsia pacifica*	Block‐type	Single‐particle cryo‐EM at 3.5 Å resolution.	16.8 MDa PBS: The model includes 862 protein subunit: comprising 4 core linkers, 16 rod‐core linkers, and 52 rod linker, and maps a total of 2048 chromophores. This structure clarifies how specific linker–phycobiliprotein interactions occur and how the linker framework is assembled.	Zhang et al. (2017)

In this review, we summarize these structural advances, highlight current understanding of photoprotective regulation, and discuss emerging biotechnological applications. Together, these perspectives underscore the importance of resolving PBS architecture at atomic detail as a foundation for future bioengineering efforts, including the design of cyanobacterial cell factories.

## GENERAL ARCHITECTURE: ANTENNA BUILDING BLOCKS

**Figure 1:**
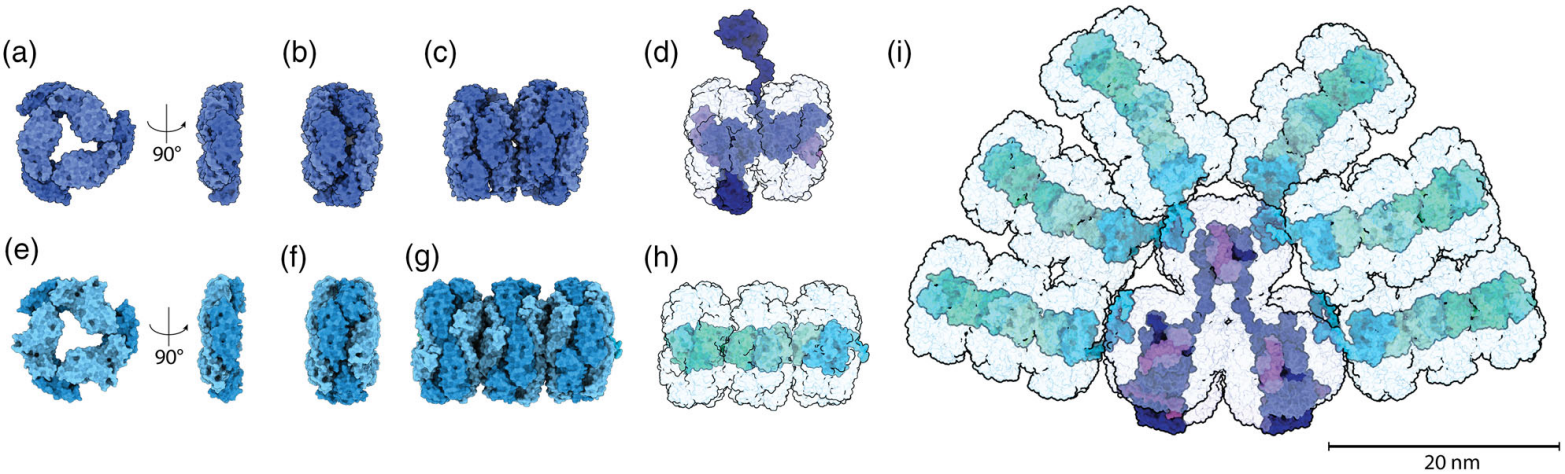
General building blocks of phycobilisomes (PBSs) illustrated using the hemidiscoidal type. (a) Disc‐like allophycocyanin (APC) heterotrimers (αβ)_3_. (b) Face‐to‐face stacking of APC trimers forms heterohexameric APC discs (αβ)_6_. (c) APC discs stack into APC cylinders. Directional energy transfer toward the photosynthetic reaction centers is mediated by specialized terminal emitter subunits such as ApcD and ApcF (not shown for simplicity), which occupy defined positions within the APC core. (d) APC cylinders (APC (αβ)_6_ rendered transparent) are held together by core linker proteins that fill the central cavities of the APC discs and assemble the APC core architecture. (e) Disc‐like phycocyanin (PC) heterotrimers (αβ)_3_. (f) Face‐to‐face stacking of PC trimers forms heterohexameric PC discs (αβ)_6_. (g) PC discs stack into PC rods. (h) PC rods (PC (αβ)_6_ rendered transparent) are stabilized by rod‐linker proteins that fill the central cavities of the PC discs and anchor the rods at defined positions on the APC core. (i) Fully assembled PBS formed through the coordinated interactions of core and rod‐linker proteins with the APC core and PC rods. APC and PC discs are shown transparent to reveal the underlying linker ‘skeleton’. Structural elements were modeled using PBS coordinates from PDB 7SCA and 7SC9 and the corresponding cryo‐EM map EMD‐25068 (Dominguez‐Martin et al., 2022). All structures are rendered to scale. Scale bar: 20 nm.

The PBS is a giant, multi‐subunit pigment–protein complex, with a size ranging from 1.2 to 16.8 MDa (Adir et al., 2020). They are composed of chromophore‐binding PBPs and colorless proteins called LPs (de Marsac & Cohen‐bazire, 1977). In the canonical PBS structure, the PBPs and LPs assemble into a central core surrounded by radiating peripheral rods (Figure 1). The PBPs form heterodimers (α and β), with both subunits typically carrying one to three covalently attached phycobilins at conserved cysteine residues (Adir, 2005; Bryant et al., 1991), although in certain specialized subunits pigments are not covalently bound (Consoli et al., 2025). These heterodimers assemble into disc‐like trimers (αβ)_3_ (Figure 1a,e) and further into hexamers (αβ)_6_ (Figure 1b,f), which stack to form the rods (Figure 1g) and core cylinders (Figure 1c). The PBPs have a globin fold binding an open‐chain tetrapyrrole, and there are four major subgroups: allophycocyanin (APC, *λ*_max_ = 652 nm, absorb low energy), phycocyanin (PC *λ*_max_ = 620 nm, absorb intermediate energy), phycoerythrin (PE *λ*_max_ = 560 nm, absorbs high energy), and phycoerythrocyanin (PEC, *λ*_max_ = 575 nm), and the main bilins, that are open‐chain tetrapyrroles differing mainly in their pattern of double bonds and side chains, which shifts their color and absorption: phycocyanobilin (PCB *λ*_max_ = 620–630 nm), phycoerythrobilin (PEB *λ*_max_ = 550–560 nm), phycourobilin (PUB *λ*_max_ = 495 nm), and phycoviolobilin (PVB *λ*_max_ = 570–580 nm) (Table 2). LPs are mostly colorless polypeptides that bind in the central cavity of the PBPs discs forming the skeleton of the PBS (Figure 1d,h,i). They can be classified into four groups based on their locations: L_CM_ (core‐membrane), L_C_ (core), L_R_ (rods), L_RC_ (rod‐core) (Adir, 2005; Adir et al., 2020; de Marsac & Cohen‐bazire, 1977; Liu et al., 2021; Zhang et al., 2017). LPs also fine‐tune the spectral properties of the chromophores bound to the PBPs, ensuring a downhill energy‐transfer pathway toward the photosynthetic reaction centers (Glazer, 1985).

**Table 2:** Summary of the typical absorption maxima (*λ*_max_) of the four principal open‐chain tetrapyrrole chromophores

Bilin	Typical *λ*_max_ (free)	Protein‐bound *λ*_max_ range	Main protein carriers
PUB	~495 nm	490–505 nm	Marine PE variants
PEB	550–560 nm	545–565 nm	PE
PVB	570–580 nm	565–590 nm	PEC
PCB	620–630 nm	615–640 nm	PC, APC

## EVOLUTION

PBSs appear to have originated very early in cyanobacterial evolution, before the major diversification of extant cyanobacterial lineages and prior to the primary endosymbiotic event that produced red algae and glaucophytes, all of which retain PBS or PBS‐derived antenna systems (Matsuo et al., 2025; Stadnichuk & Kusnetsov, 2023). Comparative genomics and pigment biosynthesis pathways suggest that the earliest PBSs were compact hemidiscoidal assemblies composed primarily of APC cores with short PC rods, enabling early phototrophs to capture wavelengths poorly absorbed by chlorophyll (Jiang et al., 2023; Zheng et al., 2021; Zilinskas & Greenwald, 1986). As cyanobacterial lineages diversified, PBSs diversified through a combination of gene duplication and divergence of PBPs, expansion of bilin lyase families that control pigment attachment and spectral tuning, and the emergence of modular LPs that stabilized rods, extended their length, and refined energy‐transfer directionality (Lee et al., 2019). These innovations allowed the incorporation of additional pigments such as PEB and PUB in PE, or PVB in PEC, broadening the absorption spectrum and facilitating colonization of deeper, shaded, or spectrally filtered environments. Over evolutionary time, PBS cores also became more elaborate, ranging from simple cylinders to tricylindrical and pentacylindrical architectures (Jiang et al., 2023), enabling adaptation to diverse ecological niches, with red algae often exhibiting the most complex structures (Ma et al., 2020; Zhang et al., 2017). Despite the broad evolutionary framework that has emerged, substantial uncertainties remain. The precise sequence of molecular and structural innovations leading to modern PBSs is still unresolved, the evolutionary origin of LPs remains obscure, the architecture of the ancestral complex cannot be reconstructed with confidence, and the role of horizontal gene transfer in shaping pigment diversity continues to be debated. Nevertheless, the overall picture is clear: PBSs emerged as highly adaptable light‐harvesting systems that enabled early oxygenic phototrophs to exploit wavelengths poorly absorbed by chlorophyll, thereby accessing a broader spectral range for photosynthesis, one that likely dominated in Archaean oceans (Matsuo et al., 2025).

## ARCHITECTURE AND DIVERSITY OF PHYCOBILISOMES

**Figure 2:**
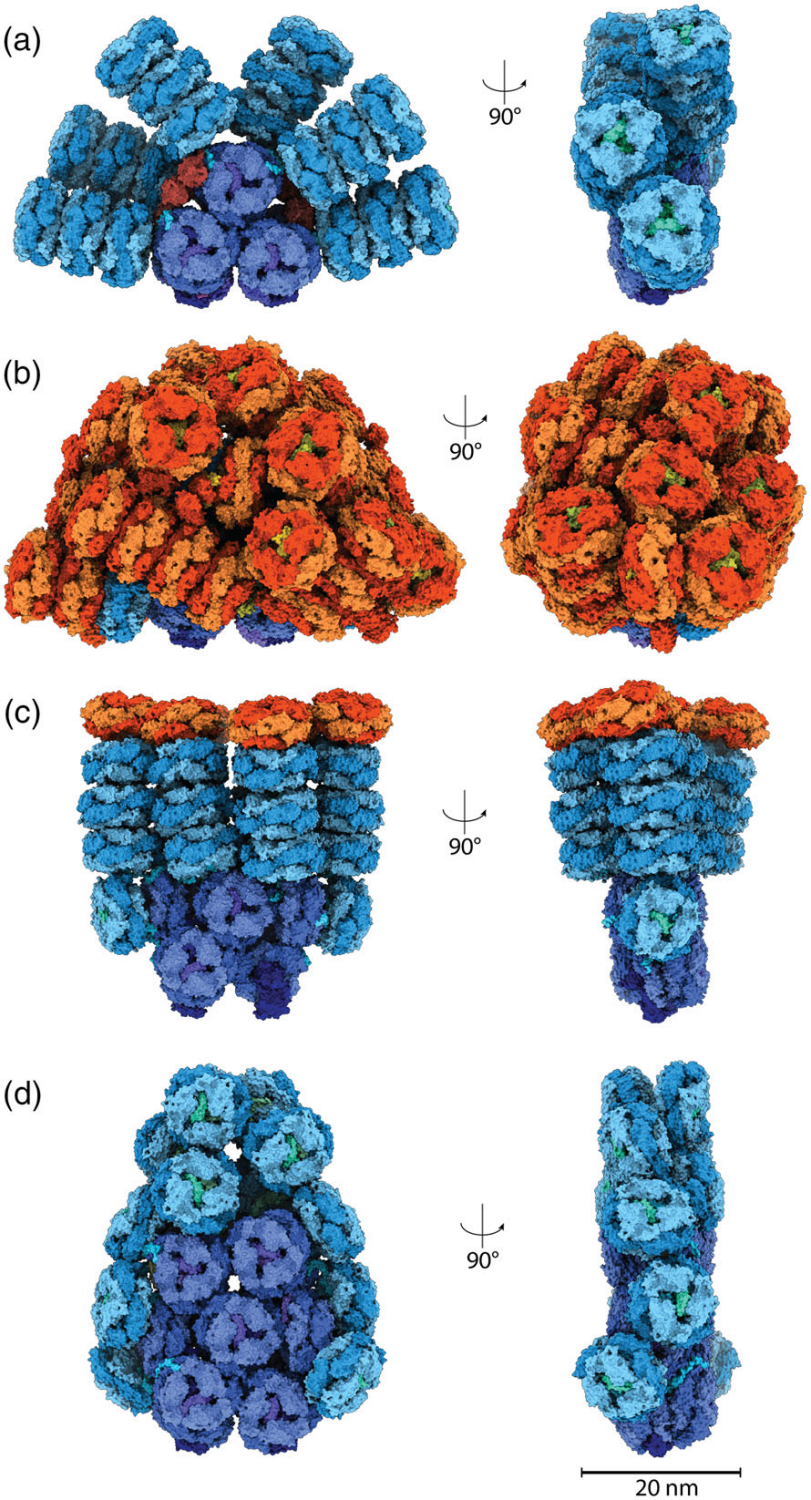
Four main phycobilisome (PBS) architectures. Representatives of each architecture are shown: allophycocyanin (APC) cores in blue‐purple hues, phycocyanin (PC) rods in blue‐cyan, and phycoerythrin (PE) rods in orange. (a) Hemidiscoidal PBSs found in cyanobacteria represented by the PBS from *Synechocystis* sp. PCC 6803 (PDB: 7SCA, 7SC9; EMDB: 25068). They are the most versatile PBS structures. (b) Hemiellipsoidal PBSs are the biggest PBSs found in red algae, represented by the PBS from *Porphyridium purpureum* (PDB: 6KGX). (c) Bundle‐shaped PBSs are found in *Gloeobacter* species, early‐branching cyanobacteria that lack thylakoid membranes. Bundle‐type PBSs possess rods that are at least 2–3 discs longer than depicted as they were not well resolved in cryogenic electron microscopy (cryo‐EM) maps (Ma et al., 2025) (PDB: 9K9W, extending the rods by two discs using EMD‐64828). (d) Paddle‐shaped PBSs are characteristic of *Anthocerotibacter panamensis*, also a thylakoid‐less, early‐branching cyanobacterium. Paddle‐shaped PBSs contain four additional PC discs above those shown (Jiang et al., 2023), which were not well resolved in the available cryo‐EM maps. (PDBs: 8IMI, 8IMJ, 8IMK, 8IML, 8IMM, 8IMN, 8IMO). All structures are rendered to scale. Scale bar: 20 nm.

PBSs exhibit remarkable architectural diversity across taxa, reflecting evolutionary adaptations to distinct ecological niches and light environments. Here, we streamline previous classification schemes and propose four primary architectural classes of PBSs (Figure 2):

*Hemidiscoidal*: typically found in cyanobacteria, with bi‐, tri‐, or pentacylindrical core configurations.

*Hemiellipsoidal*: the largest PBS type, characteristic of red algae. We include the block‐shaped PBS within this category, as it is closely related and differs primarily in the length and arrangement of certain rods rather than in overall architecture.

*Bundle‐shaped*: characteristic of *Gloeobacter violaceus* and potential other *Gloeobacter* species, deeply diverged cyanobacteria that lack thylakoids; the PBS forms tall, bundled stacks of PC and PE hexamers.

*Paddle‐shaped*: recently described in *Anthocerotibacter panamensis* (Jiang et al., 2023), a thylakoid‐free cyanobacterium; features a heptacylindrical APC core and attachments of PC hexamers instead of larger PC rod assemblies.

### Hemidiscoidal phycobilisomes

Among the various morphological types of PBSs, the hemidiscoidal form is found in cyanobacteria. These supramolecular complexes are remarkable for their size, complexity, and efficiency, often exceeding 5 MDa in mass (Bryant & Gisriel, 2024). The hemidiscoidal PBS is characterized by a central core composed of stacked APC cylinders and radiating peripheral rods made of PC and, in some cases, PE or PEC (Bryant et al., 1979).

**Figure 3:**
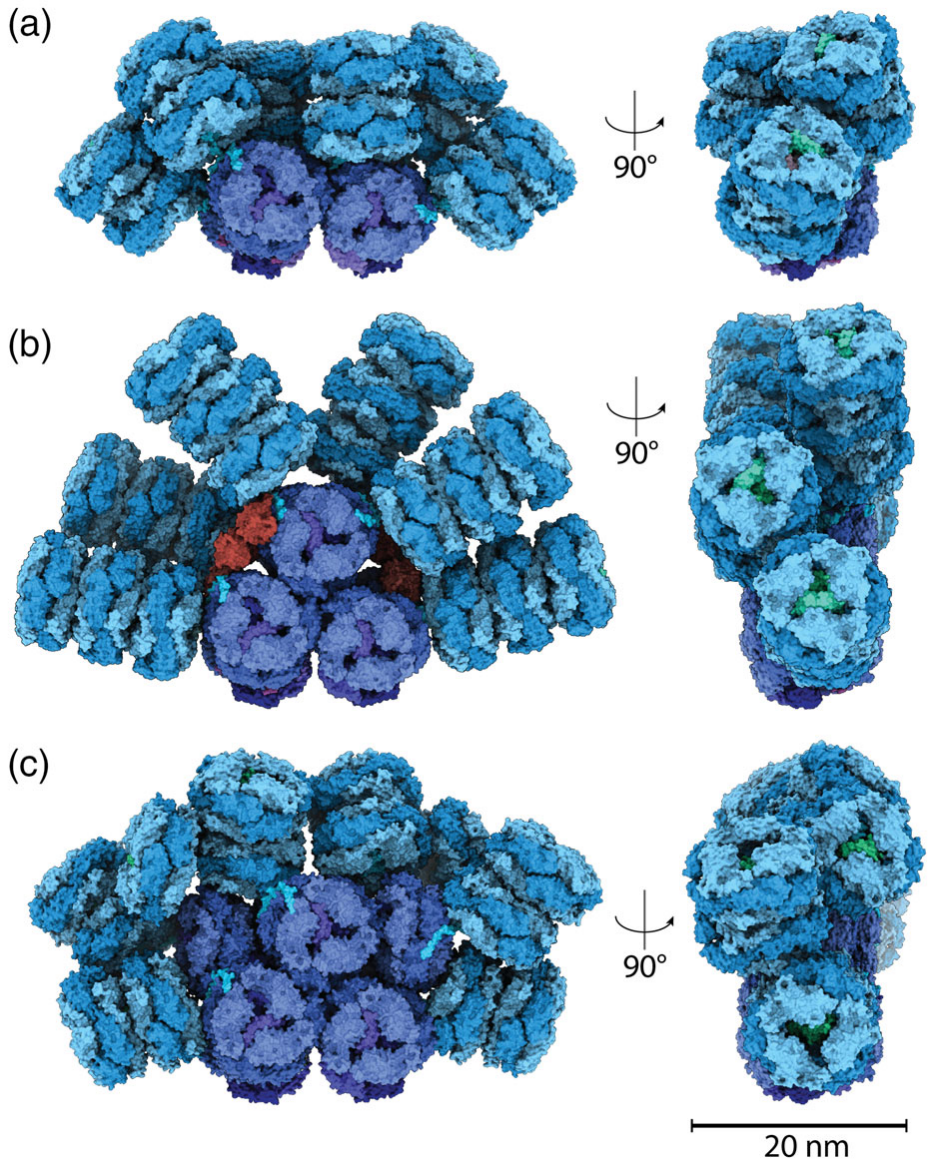
The three sub‐architecture classes of hemidiscoidal phycobilisomes (PBSs). (a) The bicylindrical PBS from *Synechococcus elongatus* PCC 7942 (PDB: 9WG7). As the name indicates, bicylindrical PBSs contain two allophycocyanin (APC) core cylinders. (b) The tricylindrical PBS from *Synechocystis* sp. PCC 6803 in complex with orange carotenoid protein (OCP) in red (see Section ‘Photoprotection’) (PDB: 7SCA, 7SC9; EMDB: 25068), which contains an additional APC cylinder that stacks on top. (c) The pentacylindrical PBS from *Anabaena* sp. PCC 7120 (PDB: 7EYD), which possesses two additional small APC cylinders positioned on either side of the top central APC cylinder. Rod length and phycocyanin (PC)/phycoerythrin (PE) composition can vary depending on the species. All structures are rendered to scale. Scale bar: 20 nm.

At the foundation are the APC trimers, each composed of α‐subunits (ApcA) and β‐subunits (ApcB) that bind PCB chromophores through conserved cysteine residues (Sonani et al., 2015). APC trimers spontaneously assemble into disk‐like (αβ)_3_ units, which then stack into cylinders. These cylinders form the basal and upper layers of the core, with their relative orientation and spacing tightly controlled to maintain optimal chromophore distances for ultrafast energy transfer. Regardless of the hemidiscodial PBS architecture, there are two APC cylinders in the basal layer, either no, one, or three additional APC cylinders are stacked on top of these basal APC cylinders, forming the bicylindrical, tricylindrical, or pentacylindrical architecture, respectively (Dominguez‐Martin et al., 2022; Zheng et al., 2021, 2025) (Figure 3).

The structural integrity and functional specialization of the APC cylinders are defined by a family of core LPs that act as molecular scaffolds. Among these, ApcE (also known as the core‐membrane linker or L_CM_) is the most prominent. ApcE is a large, multidomain protein that occupies a central position at the membrane‐proximal side of the core. Its extended helical arms and chromophore‐binding domains interact with multiple APC trimers, effectively locking the basal cylinders into a precise configuration (Dominguez‐Martin et al., 2022; Zheng et al., 2021, 2025). Besides serving as a key structural role, ApcE also fulfills a functional element within the basal layer; its chromophore is red‐shifted relative to the bulk APC cylinders, functioning as excitation energy funnel toward the terminal emitters (Assefa et al., 2024; Chang et al., 2015; Elanskaya et al., 2018; Stadnichuk & Krasilnikov, 2024; Zheng et al., 2021). The chromophores in the specialized terminal emitters ApcD and ApcF exhibit the most pronounced red shifts in the core and thus sit at the end of the energy‐transfer cascade, accepting excitation from ApcE and the surrounding APC trimers and delivering it directly to the photosynthetic reaction centers (Calzadilla et al., 2019; Dominguez‐Martin et al., 2022; Dong et al., 2009; Zheng et al., 2021). Together, this hierarchy of chromophore red shifts ensures a strictly downhill, highly efficient flow of excitation energy from the peripheral rods to the photosystems.

The assembly process is thought to proceed through a series of modular steps, although the precise temporal order has not been experimentally resolved. APC trimers form spontaneously, guided by the intrinsic folding properties of ApcA and ApcB and the action of bilin lyases that attach the chromophores (Bodunova et al., 2025; Liu et al., 2010). These trimers then associate into cylinders. The incorporation of ApcE defines the orientation of the basal layer and anchors the entire structure to the thylakoid membrane (Ajlani et al., 1995; Chang et al., 2015; Elanskaya et al., 2018).

Through this combination of modular APC trimers, precisely positioned LPs, and a sequential assembly pathway, the APC core achieves a remarkable balance of mechanical stability, structural symmetry, and photophysical efficiency. The resulting architecture is not merely a passive scaffold but an active energy‐processing machine, optimized through evolution to capture, funnel, and deliver excitation energy with near‐quantum efficiency. As recent cryo‐EM studies continue to reveal new details of core organization (Dominguez‐Martin et al., 2022; Kawakami et al., 2022; Zheng et al., 2021, 2025), it is becoming increasingly clear that the APC core represents one of the most elegant examples of supramolecular assembly in photosynthetic light‐harvesting systems.

In hemidiscoidal PBSs, the rods form a highly ordered, radially symmetric array of PBPs stacks whose architecture is defined by both, the number and composition of rod cylinders, and the extensive network of rod‐specific LPs that enforce the characteristic half‐disc geometry. Each rod typically consists of linear stacks of PC hexamers, often arranged as (αβ)_6_ disks that chain together through interactions mediated by CpcC‐type rod linkers (de Lorimier, Guglielmi, et al., 1990; Ughy & Ajlani, 2004). In many red algae and some cyanobacteria, the distal segments of the rods incorporate PE introducing additional bilin types (PEB and/or PUB) (Six et al., 2005, 2007) that extend the absorption cross‐section and create a finely graded energy ladder toward the core. The rod architecture is stabilized by a hierarchical set of LPs: CpcC (rod linker), CpcD (rod cap), CpcG (rod‐core linker), and species‐specific rod linkers that define rod length and the spacing between adjacent PC disks. CpcC binds at the interface of PC hexamers, imposing the correct axial rise and rotational offset required for efficient excitonic coupling (Dominguez‐Martin et al., 2022), while CpcD caps the distal end of the rod, preventing uncontrolled polymerization (de Lorimier, Bryant, & Stevens Jr., 1990) and tuning the distal bilin environment (Zheng et al., 2023). The rod‐core junction is mediated by CpcG, a long, acidic linker that replaces the canonical CpcD cap on the proximal PC disk and provides the docking interface for the core cylinders. Hemidiscoidal PBSs often encode multiple CpcG paralogs, each conferring distinct rod‐core geometries and enabling species‐specific variations in rod number and orientation (Bryant et al., 1979; Dominguez‐Martin et al., 2022; Kawakami et al., 2022; Zheng et al., 2021). Spectroscopically, the rods exhibit ultrafast energy transfer along the PC stacks (1–5 ps), with distal PE segments transferring energy inward via red‐shifted bilins and linker‐induced conformational rigidity that minimizes energy loss (David et al., 2014; Hirota et al., 2021; Sil et al., 2022; Sohoni et al., 2023).

The rods of hemidiscoidal PBSs exhibit substantial evolutionary and physiological diversification that modulate light‐harvesting performance across ecological niches. A central feature of this diversification is chromatic acclimation (CA), a regulatory process that adjusts the relative amounts and types of PBPs incorporated into the rods in response to ambient light color (Hirose et al., 2019; Sanfilippo et al., 2019). These acclimation strategies illustrate the remarkable regulatory flexibility of cyanobacteria in reshaping their light‐harvesting antennae, with light‐responsive systems such as CcaS/CcaR (Hirose et al., 2017) directly modulating pigment composition, bilin content, and the spectral properties of hemidiscoidal PBS rods.

Recently, cryo‐EM shows that hemidiscoidal PBSs can adopt multiple global conformations (e.g., ‘up–up’ and ‘down–down’ states), reflecting pivoting of entire rod assemblies relative to the APC core (Dominguez‐Martin et al., 2022). This global mobility is enabled by tier‐specific flexibility: Rods attached to the upper APC layer exhibit greater angular freedom than those anchored to the basal layer (Dominguez‐Martin et al., 2022; Ma et al., 2020). The functional significance of these distinct conformational states remains unclear, especially because they introduce only subtle changes in excitation flow toward the terminal emitters (Dominguez‐Martin et al., 2022). Nevertheless, their existence suggests that PBSs may exploit a degree of structural plasticity for purposes other than optimizing steady‐state energy transfer. One possibility is that these conformations modulate how the PBS docks onto PSI or PSII, transiently adjusting interaction geometry under fluctuating light. Alternatively, conformational flexibility may facilitate mechanical resilience, allowing the PBS to absorb photophysical or osmotic stresses without compromising its overall architecture.

These evolutionary and regulatory layers underscore that hemidiscoidal rods are not static scaffolds but highly adaptable, compositionally tunable antenna modules, capable of extensive remodeling to optimize energy capture and transfer under fluctuating environmental conditions, properties that other PBS architectures cannot match.

#### Rod‐shaped PSI antenna

**Figure 4:**
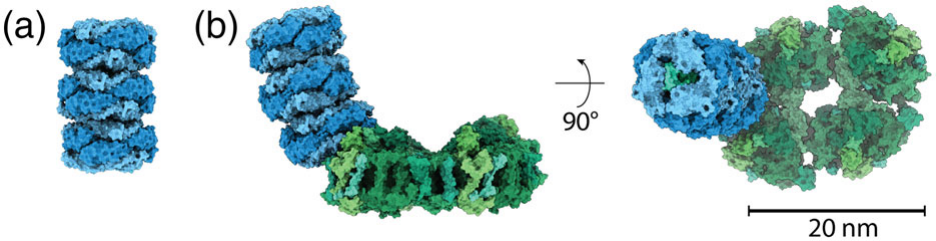
Rod‐shaped dedicated photosystem I (PSI) antenna. The rods of the phycobilisomes (PBS) can be repurposed as a dedicated PSI antenna in some species under certain environmental conditions using a specialized linker CpcL. (a) Phycocyanin (PC) rod that associates with PSI, structurally identical to a standard PBS rod except that CpcL replaces the canonical rod‐core linker CpcG. (b) Model of the CpcL‐type rod (cyan colors) attached to PSI (green colors), generated using structural information from PDB entries 9W4J and 9WD5, as well as cryogenic electron microscopy (cryo‐EM) map EMD‐65883 (Mao et al., 2026). All structures are rendered to scale. Scale bar: 20 nm.

An intriguing feature of some hemidiscoidal PBSs is that a single PBS rod can dock directly onto PSI, thereby expanding its effective absorption cross‐section (Figure 4). This interaction is mediated by a specialized paralog of the canonical rod‐core linker CpcG, historically termed CpcG2 or CpcG3, which diverges from other CpcG family members by carrying a hydrophobic C‐terminal α‐helical extension capable of anchoring the rod to the thylakoid membrane (Mao et al., 2026). The antenna complex is an ~800‐kDa rod roughly 160 Å long and 100 Å in diameter, typically comprising three linearly arranged PC hexamers (Mao et al., 2026; Zheng et al., 2023), closely resembling the rods found within the PBS. Functional studies in *Synechocystis* sp. PCC 6803 demonstrated that PC associated with CpcG1 primarily transfers energy to PSII, whereas PC bound to CpcG2 preferentially excites PSI (Kondo et al., 2007). Because of these unique properties, CpcG2/CpcG3 was renamed CpcL, reflecting its role as a linker that creates a PSI‐specific PC antenna rather than a component of the hemidiscoidal PBS (Watanabe & Ikeuchi, 2013). Each rod contains one copy of CpcL, the rod‐linker pair CpcC1 and CpcC2, and a single FNR molecule bound via its CpcD‐like domain; the catalytic FNR domain remains flexible and was not fully resolved (Zheng et al., 2023). Spectroscopic analyses show that these rods absorb maximally at 620 nm and emit at 670 nm, consistent with efficient PC to PSI energy transfer.

Recent work has clarified the physiological relevance of these complexes. Under iron starvation, *Anabaena* sp. PCC 7120 forms exceptionally tight PSI–CpcL–PC–FNR supercomplexes, including stable assemblies with PSI monomers and dimers (Shimizu et al., 2023). Interestingly, PSI tetramers did not form analogous complexes under iron‐depleted conditions, in contrast to earlier observations under iron‐replete growth (Watanabe et al., 2014), suggesting that the PSI oligomeric state and membrane composition modulate CpcL‐rod binding.

The architecture of these rod‐shaped complexes highlights the evolutionary plasticity and modularity of cyanobacterial light‐harvesting systems, demonstrating how structural elements originally belonging to the peripheral rods of hemidiscoidal PBSs can be redeployed and reconfigured into a standalone, PSI‐directed antenna with distinct photophysiological functions.

#### Helical allophycocyanin nanotubes

**Figure 5:**
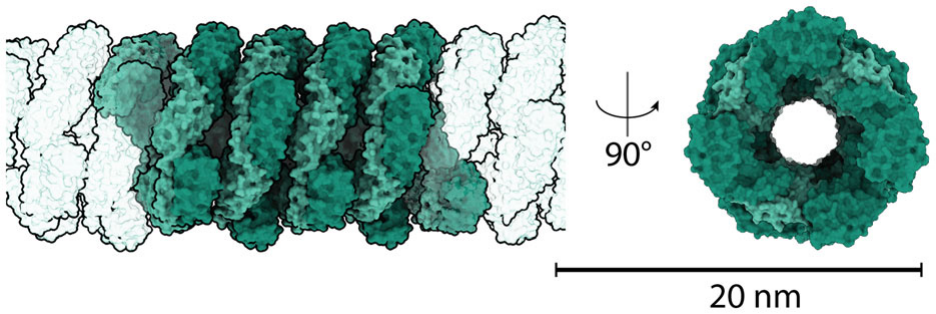
Helical allophycocyanin (APC) nanotube. Specialized APC alpha and beta subunits assemble into a filamentous structure instead of heterohexamer (alpha/beta) 6 like regular APC alpha and beta subunits. The central cavity is hypothesized to be filled out by the C‐terminal extension of IsiX and binding it to photosystem I (PSI) (Gisriel et al., 2023) (PDB: 8DDY). Structures are rendered to scale. Scale bar: 20 nm.

The recent cryo‐EM characterization of APC nanorods has revealed a previously unrecognized extension of the cyanobacterial light‐harvesting apparatus, adding a new architectural element to the classical PBS framework (Gisriel et al., 2023). These APC nanorods form elongated, linear stacks of specialized APC α‐ and β‐subunits, creating a filamentous structure (Figure 5). Although the exact length likely varies among species and physiological conditions, the reconstructions clearly demonstrate that APC can form extended rod‐like assemblies likely much longer than APC cylinders within PBS cores.

The pigment environment within these nanorods produces red‐shifted absorption properties, positioning them as efficient collectors of long‐wavelength far‐red light. Based on their spectral characteristics and spatial organization, Gisriel et al. (2023) propose that APC nanorods primarily harvest light for PSI, effectively extending PSI excitation under low‐light and spectrally shifted conditions. Energy‐transfer analyses support this interpretation, revealing a continuous excitonic pathway along the nanorod axis that is well suited for directing excitation toward PSI with minimal loss (Gisriel et al., 2023).

The existence of APC nanorods expands the known diversity of cyanobacterial antenna architectures and demonstrates that APC, long considered a core‐restricted PBP, can be repurposed into extended light‐harvesting elements and suggests that cyanobacteria have evolved additional, previously unrecognized strategies for tuning their antenna systems to specific spectral niches. The discovery of APC nanorods therefore provides not only a new structural framework for understanding far‐red light harvesting but also a compelling example of the evolutionary plasticity of cyanobacterial antenna systems.

#### Photoprotection

Exploiting sunlight for photosynthesis is essential for cyanobacterial growth, yet excessive excitation can become harmful by driving the formation of ROS and ultimately causing cellular damage. To prevent this, photosynthetic organisms have evolved non‐photochemical quenching (NPQ) mechanisms that safely dissipate surplus absorbed energy. In cyanobacteria with hemidiscoidal PBSs, NPQ is uniquely mediated by the orange carotenoid protein (OCP), whose activity depends strictly on interaction with the APC core (Figure 3b) (Dominguez‐Martin et al., 2022; Sauer et al., 2024; Wilson et al., 2006). Under intense blue‐green light, OCP undergoes carotenoid‐driven activation and transitions into its red, active form, which binds to the PBS core at the ApcA/ApcB interface, inducing rapid dissipation of excitation energy as heat (Kirilovsky & Kerfeld, 2013; Leverenz et al., 2015). Restoration of full antenna function is regulated by the fluorescence recovery protein (FRP), which accelerates OCP deactivation and facilitates its release from the PBS, thereby re‐establishing efficient energy transfer to the photosystems (Boulay et al., 2010; Gwizdala et al., 2011). In addition to this canonical OCP‐FRP cycle, many cyanobacteria encode helical carotenoid proteins (HCPs), homologous to the OCP N‐terminal domain, that can also quench PBS‐derived excitation energy, pointing to a broader and more diversified carotenoid‐based photoprotective system within lineages that retain hemidiscoidal PBS architecture (Muzzopappa & Kirilovsky, 2020; Sheppard et al., 2025).

The hemidiscoidal PBS stands out among light‐harvesting antennae for its exceptional architectural flexibility, a property rooted in its modular design: an APC core that provides a stable scaffold and peripheral rods that can be compositionally, structurally, and functionally reconfigured with remarkable ease. This modularity enables extensive diversification in rod pigments, LPs, and chromophore chemistries across cyanobacterial lineages, while also supporting dynamic physiological remodeling through CA, nutrient‐responsive rod degradation, and conformational switching of entire rod tiers. The same design allows individual rods to be repurposed into dedicated PSI antennas. Together, these features reveal the hemidiscoidal PBS as a uniquely adaptable antenna system capable of tuning its composition, structure, and interactions to optimize light harvesting, photoprotection, and membrane organization across an exceptionally wide range of ecological and environmental conditions.

### Hemiellipsoidal phycobilisomes

**Figure 6:**
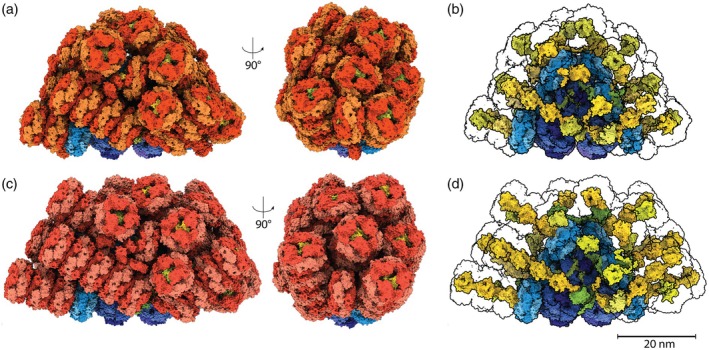
Red algae hemiellipsoidal phycobilisomes (PBSs). (a) Hemiellipsoidal PBS from *Porphyridium purpureum* (PDB: 6KGX). Allophycocyanin (APC) cores are shown in blue‐purple hues, phycocyanin (PC) rods in blue‐cyan, and phycoerythrin (PE) rods in orange. (b) Transparent B‐phycoerythrin (B‐PE) discs (phycoerythrobilin [PEB]/phycourobilin [PUB]‐containing) reveal the underlying rod‐linker proteins, PC discs, and APC core. (c) Block‐shaped PBS from *Griffithsia pacifica* (PDB: 5Y6P). Despite its distinct outer geometry, its overall organization is highly similar to the hemiellipsoidal PBS of *P. purpureum* and can be viewed as a sub‐architecture of the hemiellipsoidal type. The slightly different coloration of the outer discs reflects the higher PUB content in the *G. pacifica* PBS. (d) Transparent R‐phycoerythrin (R‐PE) discs highlight the rod‐linker proteins, PC discs, and APC core. The inner PBS architecture is nearly identical to that of *P. purpureum*. All structures are rendered to scale. Scale bar: 20 nm.

Hemiellipsoidal PBSs constitute one of the most structurally elaborate and functionally optimized light‐harvesting architectures found in red algae, distinguished by their asymmetric half‐ellipsoid geometry (Figure 6). The hemiellipsoidal PBSs from *Porphyridium purpureum* possess a tricylindrical APC core similar to tricylindrical hemidiscodial PBSs (Gantt, 1996; Ma et al., 2020). This core is stabilized by the core‐membrane linker L_CM_ (ApcE), small core linkers such as L_C_ (ApcC), and a suite of rod‐specific linkers that tune excitonic coupling and organize the PE‐ and PC‐rich rod segments (Ma et al., 2020). From this, APC core radiates 14 peripheral rods, seven on each side, each composed of stacked PC or PE hexamers (Ma et al., 2020). Their organization and curvature are imposed by a hierarchical set of rod linkers that generate the characteristic hemiellipsoidal geometry. They include L_RC_‐type linkers that anchor rods to the core, L_R_‐type linkers that connect inner PC discs but also attach specialized rods and L_Rγ_‐type linkers that bind the outer B‐phycoerythrin (B‐PE) discs to the rods. These distal rod segments contain PUB contributing to the characteristic coloration of the *P. purpureum* PBS. The proximal rod segments are enriched in PC, establishing a finely graded spectral ladder that funnels excitation energy efficiently toward the multi‐cylindrical APC core. The terminal emitter, primarily ApcE (L_CM_), together with ApcD and ApcF, forms the final energy‐transfer interface with PSII. The bilin environment within ApcE acts as a major spectral tuner, defining the terminal emission wavelength and ensuring efficient energy funneling from the PE‐ and PC‐rich rods into the APC core. Although ultrafast spectroscopy has not yet been applied to intact hemiellipsoidal PBSs, their structural organization strongly suggests the capacity for picosecond‐scale, highly directional energy transfer. The precise chromophore spacing, ordered linker scaffolding, and the presence of red‐shifted terminal bilins in ApcE and ApcD (Ma et al., 2020) mirror features known to support ultrafast excitation flow in hemidiscoidal cyanobacterial PBSs, implying similar principles may operate in the hemiellipsoidal architecture.

The block‐shaped PBS isolated from *Griffithsia pacifica* (Zhang et al., 2017) is closely related to the hemiellipsoidal PBS of *P. purpureum* and is therefore best considered a sub‐type rather than a distinct architectural class (Figure 6c,d). It exhibits a compact, rectilinear organization in which the APC core and rods form a quasi‐rectangular block, differing from the *P. purpureum* hemiellipsoidal PBS mainly in its modest variation in rod length.

The core of the *G. pacifica* PBS is a multi‐cylindrical APC assembly that is nearly identical to the tricylindrical APC core of *P. purpureum* (Figure 6b,d), and the rods are arranged onto the core in an analogous manner. However, several of the *Griffithsia* rods are slightly longer, and their distal segments contain PUB‐rich R‐phycoerythrin (R‐PE) rather than the PEB‐rich B‐PE found in *P. purpureum*, resulting in slightly different absorption properties (Figure 6) (Xie et al., 2021).

Functionally, the block‐shaped PBS of *Griffithsia* appears to represent a red‐algal specialization optimized for stable, blue‐green‐shifted subtidal light environments. However, the broader distribution of block‐type PBSs across red algae remains underexplored.

The evolutionary diversification of hemiellipsoidal PBSs reflects long‐standing selective pressures on chromophore composition and linker‐mediated supramolecular organization, producing exceptionally large light‐harvesting antennae well suited to the blue‐green shifted, often low‐light environments inhabited by many red algae.

Recent structural work has shown that hemiellipsoidal PBSs adjust their architecture in response to light availability, shedding four rod discs from the top of the structure under medium‐light compared with low‐light conditions, resulting in an overall ~9% reduction in pigment content (Dodson et al., 2023). However, compared to the highly modular hemidiscoidal PBSs of cyanobacteria, red‐algal hemiellipsoidal PBSs show only limited capacity for pigment remodeling in response to environmental change. To date, no CA or comparable rod‐level pigment switching has been demonstrated in red algae, implying that their architecture is substantially more constrained. Instead, regulatory responses appear to operate primarily at the level of PBS mobility and spatial reorganization on the thylakoid membrane, processes documented in red algae such as *Porphyridium* and *Cyanidioschyzon* (Kana et al., 2014), although the full extent of such mechanisms has not been systematically explored across the group.

Genomic analyses of red algae reveal extensive diversification of PBS linker families, including lineage‐specific expansions of PE‐associated linkers such as CpeC, CpeD, CpeE, and CpeF (Bhattacharya et al., 2013; Matsuzaki et al., 2004; Tang et al., 2023), consistent with broader comparative genomic evidence showing large‐scale duplications of PBS linker gene families in mesophilic red algae (Lee et al., 2019). This expanded linker repertoire, exceeding the subset of linkers resolved in the two currently available red‐algal PBS structures, suggests that additional PBS architectures or at least further structural variants likely exist within red algae.

### Bundle‐shaped phycobilisome

Bundle‐shaped PBSs represent a unique and likely ancestral light‐harvesting architecture found exclusively in members of the genus *Gloeobacter*. One representative, *G. violaceus* PCC 7421, was originally isolated from wet limestone surfaces in Switzerland (Rippka et al., 1974). Its cells display a striking violet pigmentation due to their unusually low chlorophyll *a* content and the dominance of PBPs, including APC, PC, and a distinctive PE that also carries PUB.

The recent cryo‐EM studies represent a major advance in our understanding of bundle‐shaped PBSs (Burtseva et al., 2025), providing the first near‐atomic structural insights into the rod architecture and photoprotective mechanisms of *G. violaceus* PCC 7421 (Figure 2c). As the earliest‐branching cyanobacterial lineage and the only group that entirely lacks thylakoid membranes, *Gloeobacteria* have long been hypothesized to retain ancestral features of PBS organization, yet structural information has remained fragmentary for decades.

The structural data reveal that five APC cylinders form a central core‐like scaffold to which the rods attach, consistent with earlier biochemical work suggesting a pentacylindrical APC core (Guglielmi et al., 1981; Koyama et al., 2006). The cryo‐EM data now uncover specific linker‐binding surfaces that mediate rod‐core docking by two genus unique LPs L_RC_^91kDa^ and L_RC_^81kDa^ encoded by *Glr1262* and *Glr2806*, respectively. Each L_RC_^91kDa^ anchors three rods to the APC core in a triangular arrangement forming the characteristic rod bundles instead of the radiating rods in hemidiscodial PBSs (Figure 2c). The lateral rods are attached to the APC core each by a L_RC_^81kDa^ (Burtseva et al., 2025; Ma et al., 2025).

The arrangement of bilin chromophores within the rod reveals a steep, directional energy gradient, with terminal PC bilins positioned to funnel excitation toward the APC core. This organization suggests that even in the absence of thylakoid membranes, *Gloeobacter* maintains a highly optimized energy‐transfer network capable of delivering excitation efficiently to the photosystems embedded directly in the plasma membrane.

Interestingly, rod length varies in *G. violaceus* depending on the growth conditions, indicating that bundle‐type PBSs can remodel their antenna size in response to environmental cues (Ma et al., 2025). Although the exact number of hexameric discs in each rod under different growth conditions has yet to be structurally resolved, PBS particles isolated from different light regimes show elongated rods under low‐light conditions. The extensions are likely PE‐derived, consistent with the pronounced absorption differences observed between the two growth conditions (Ma et al., 2025). This suggests that PE contributes both to rod elongation and to tuning the antenna to changes in light quality, highlighting rod‐length modulation as an ancestral adaptive feature of PBSs.

*Gloeobacter violaceus* encodes two divergent OCP3a paralogs, OCP3a_α and OCP3a_β, both of which bind exclusively echinenone and are photoactive. Spectroscopic analyses showed that photoactivated OCP3a_α induces strong and rapid quenching of the APC core, whereas OCP3a_β quenches more slowly and less efficiently. This functional divergence indicates that the pentacylindrical PBS core contains two distinct OCP‐binding sites: one likely analogous to the OCP‐binding site characterized in the hemidiscoidal PBS of *Synechocystis* and a second site unique to pentacylindrical PBS cores. The additive quenching observed when both paralogs are present confirms that the two sites are noncompetitive. These findings demonstrate that bundle‐type PBSs possess a dual‐site OCP regulatory system, enabling flexible and potentially more nuanced photoprotection in the absence of thylakoid‐based regulatory mechanisms.

### Paddle‐shaped phycobilisome

Paddle‐shaped PBSs represent one of the most striking departures from canonical cyanobacterial antenna architecture and provide an important window into the early evolution of PBS organization (Jiang et al., 2023). Their recently discovered form in *A. panamensis*, a thylakoid‐less member of the Gloeobacteria, suggests that this lineage retains features that predate the emergence of the large, rod‐stacked hemidiscoidal PBSs characteristic of most cyanobacteria. A 2.9 Å single‐particle cryo‐EM structure (Jiang et al., 2023) revealed that the *A. panamensis* PBS is built around a heptacylindrical APC core, an architecture not previously observed in any organism. The lower five cylinders form a pentacylindrical subsubstructure, whereas the upper two cylinders constitute a distinct, additional tier connected by two copies of a novel linker, ApcH, whose tandem REP domains echo those of the membrane‐associated linker ApcE. These two core regions also differ in their APC composition: The upper cylinders contain the ApcA1‐ApcB1 isoform, while the lower five incorporate the canonical ApcA2‐ApcB2 variant. Core termination is similarly specialized, with ApcC1 capping the upper cylinders and ApcC2 capping the lower five, and the complex notably lacks the terminal emitters ApcD and ApcF, features that together underscore its deep divergence from classical cyanobacterial PBSs.

The periphery of the paddle‐shaped PBS reinforces this evolutionary distinctiveness. Instead of forming stacked PC rods, *A. panamensis* decorates each side of the APC core with a sparse but heterogeneous set of PC attachments: a single PC hexamer linked by CpcG, two additional unstacked hexamers attached via the novel linker CpcJ, and a chain of five unstacked PC hexamers extending from each upper cylinder through the action of another newly identified linker, CpcN. In total, 16 PC hexamers surround a core. This arrangement creates broad PC‐APC contact surfaces but lacks the ordered rod stacking that underpins efficient excitation energy transfer in later‐evolving PBS architectures. Structural modeling and low‐temperature fluorescence spectroscopy confirm that energy transfer from the peripheral PC units, particularly those in the CpcN‐assembled chains, is inefficient, resulting in substantial PC emission despite their proximity to the APC core. Viewed in an evolutionary context, these features suggest that the paddle‐shaped PBS may represent a surviving intermediate in PBS evolution: a modular, weakly integrated antenna system that predates the emergence of the highly optimized rod‐core assemblies found in thylakoid‐bearing cyanobacteria. Its unusual combination of a vertically elongated APC core, sparse peripheral PC attachments, and inefficient PC to APC coupling is consistent with a lineage that retained ancestral traits while evolving lineage‐specific innovations within a low‐light, thylakoid‐free cellular environment.

## PHYCOBILISOME ARRAYS

**Figure 7:**
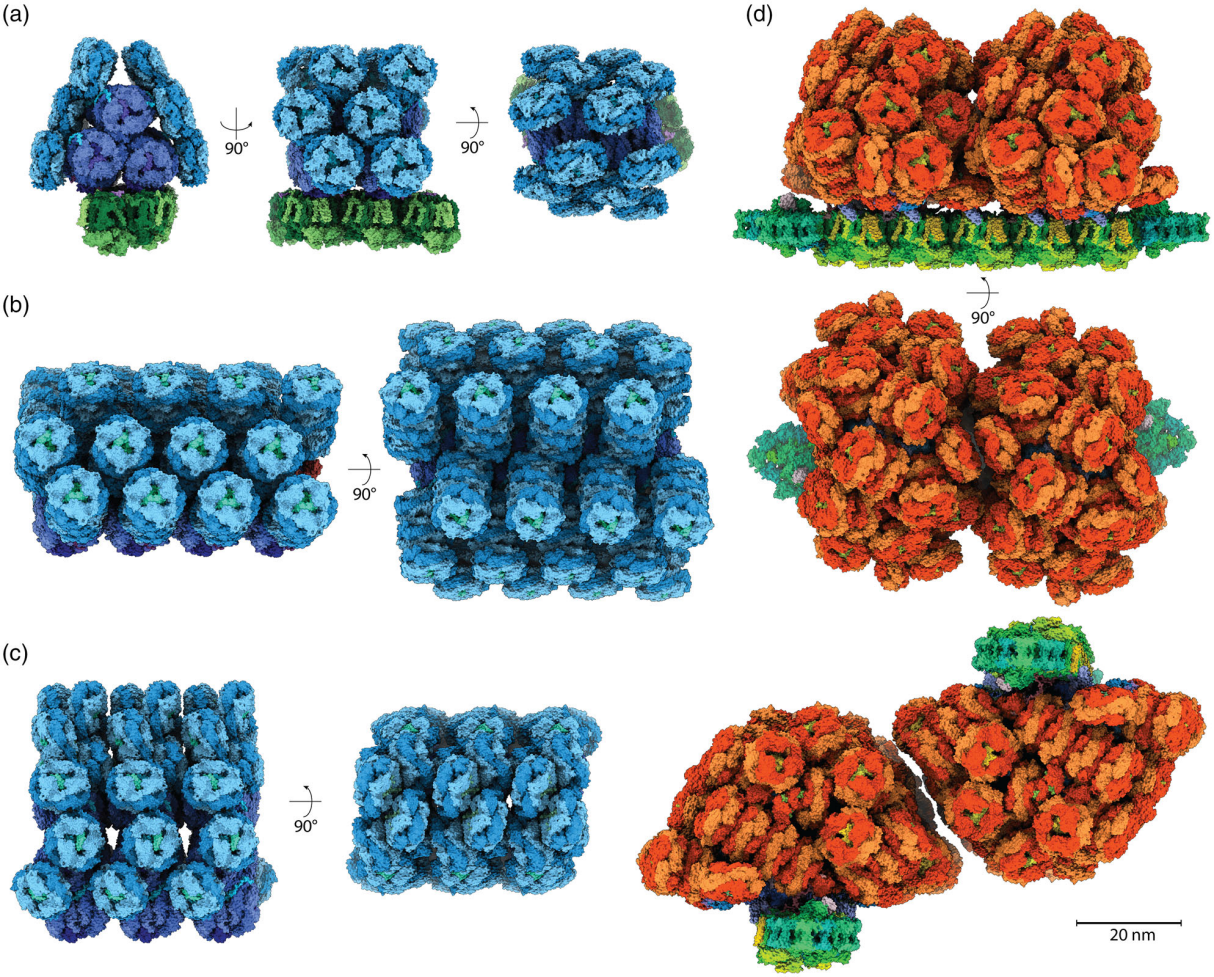
Phycobilisome (PBS) arrays. PBSs can assemble into ordered arrays that pack pigments efficiently, with individual PBSs fitting together like ‘LEGO’ pieces. (a) Two hemidiscoidal PBSs in an array arrangement in complex with photosystems II (PSIIs) from *Arthrospira* sp. FACHB439 (PDB: 8WQL); only the inner of the three rod discs is resolved. Front, side, and top views. (b) Four complete hemidiscoidal PBSs with all three rod discs resolved from *Synechocystis* PCC sp. 6803 modeled into an array (PDB: 7SCA, 7SC9; EMDB: 25068; based on EMD‐4602). Side and top views. (c) Model of an array of three paddle‐shaped PBSs from *Anthocerotibacter panamensis* (PDBs: 8IMI, 8IMJ, 8IMK, 8IML, 8IMM, 8IMN, 8IMO). Side and top views. (d) *Porphyridium purpureum* PBS cluster (PDB: 7Y7A). The PBS forms either single PBS–PSII–PSI–LHC (not shown) or double PBS–PSII–PSI–LHC megacomplexes (shown). Side and top view. These megacomplexes pack together with PBSs from the adjacent thylakoid membrane (bottom, front view), creating a tightly filled inter‐thylakoid space (modeled based on You et al. (2023)). All structures are rendered to scale. Scale bar: 20 nm.

PBSs do not function as isolated antenna particles but instead assemble into ordered, membrane‐associated arrays on the stromal surface of cyanobacterial thylakoids. Although this phenomenon was first characterized in hemidiscoidal PBSs (Adir, 2005; Rast et al., 2019), array formation appears to be a more general organizational principle shared across multiple PBS morphologies, including hemiellipsoidal (You et al., 2023), bundle‐ (Burtseva et al., 2025) and paddle‐shaped (Jiang et al., 2023) types. Early ultrastructural work revealed quasi‐regular hemidiscodial PBS lattices whose packing enhances excitation flow toward photosystems (Adir, 2005; MacColl, 1998). Cryo‐ET has substantially refined this view: Engel and colleagues demonstrated that hemidiscodial PBSs form highly organized, repeating supercomplex arrays with conserved inter‐PBS distances and defined angular alignment, tightly integrated with PSII distribution across the thylakoid membrane (Rast et al., 2019). Importantly, the close packing and defined geometry of these arrays enable excitation transfer not only within individual PBSs but also between adjacent PBS units (Dominguez‐Martin et al., 2022), enabling efficient energy transfer from antenna to photosystems within the array. Interestingly, only a single hemidiscodial PBS conformation with the mobile rods in the up–up conformation is capable of assembling into large, ordered arrays (Dominguez‐Martin et al., 2022), a constraint that may offer important clues about the functional role of the different conformations. More recently, *in situ* cryo‐ET and single‐particle analysis strategy (STAgSPA) has provided an astonishingly detailed view of PBS arrays in complex with the photosystems. Work by Zhang et al. (2024) resolved the native hemidiscoidal PBS–PSII supercomplex from *Arthrospira* sp. FACHB439 at near‐atomic resolution, revealing that PBS arrays are intimately coupled to rows of PSII dimers, forming repeating PBS–PSII modules (Figure 7a) that tile the membrane. This architecture provides a structural basis for efficient, distributed excitation delivery across multiple reaction centers. The arrays extend over large membrane regions and create a cooperative light‐harvesting surface. You et al. (2023) revealed that red‐algal hemiellipsoidal PBSs form also ordered clusters. Single and double PBS–PSII–PSI–LHC megacomplexes (Figure 7d) populate the thylakoid membrane and align with PBSs from the adjacent membrane, yielding a tightly packed, PBS‐filled inter‐thylakoid space (Figure 7d). This arrangement results in highly efficient volume usage; however, the resulting clusters are not as extensive as the large lateral arrays formed by hemidiscoidal PBS types, and it remains unclear whether excitation energy can be transferred between neighboring megacomplexes.

Although bundle‐shaped PBSs in *Gloeobacter* lack the flat geometry that enables the tight, quasi‐crystalline packing of hemidiscoidal PBSs, it could be shown that they can nevertheless form small membrane‐associated clusters (Guglielmi et al., 1981). These clusters appear less compact and less extensive than the large, ordered arrays observed in thylakoid‐bearing cyanobacteria, but they do suggest that *Gloeobacter* PBSs are, in principle, capable of limited array‐like organization. Consistent with this, Burtseva et al. recently modeled how bundle‐shaped PBSs could assemble into arrays (Burtseva et al., 2025). An intriguing feature of the paddle‐shaped PBS is that its geometry appears inherently suited for higher‐order organization on the membrane surface. The lateral width of the complex closely matches that of a PSII dimer, and the flattened, bilaterally expanded PC decorations create a regular outline that can tile across the membrane with minimal steric interference. Structural modeling (Figure 7c) suggests that the protruding PC hexamers on one PBS align with complementary recesses of the next, enabling a ‘LEGO‐like’ interlocking arrangement in which successive PBSs nest into one another very similar to hemidiscodial array formation. This modular fit would allow the complexes to assemble into extended two‐dimensional arrays, analogous to the ordered PBS–PSII supercomplex rows observed in the hemidiscodial PBS type (Zhang et al., 2024). Although *in situ* cryo‐ET data for *A. panamensis* are not yet available, TEM images reveal long, continuous rows of paddle‐shaped complexes aligned in parallel, producing extended assemblies (Jiang et al., 2023) that could support cooperative light harvesting. The ability of these putatively ancestral PBSs to form such regular arrays suggests that array formation is not a recent evolutionary innovation, but rather a more broadly accessible organizational mode that different PBS architectures can adopt to varying degrees.

Notably, OCP‐mediated NPQ can also act cooperatively across these arrays. Cryo‐EM and quantum‐chemical analysis (Dominguez‐Martin et al., 2022; Sauer et al., 2024) demonstrated that OCP binding induces pigment‐coupling changes that allow energy dissipation to propagate through neighboring PBSs, even when only a subset of PBSs carries bound OCPs. This finding highlights that PBSs and their assembly into arrays constitute a remarkably efficient architecture, optimized not only for cooperative light harvesting but also for distributed, OCP‐mediated photoprotection, allowing cyanobacteria to buffer rapid irradiance fluctuations without requiring every PBS to carry a bound OCP and thereby integrating light harvesting, energy distribution, and photoprotection into a single mesoscale network.

## EVOLUTIONARY LOGIC OF THE PHYCOBILISOME ARCHITECTURES

PBS architecture varies widely across cyanobacteria, red algae, and glaucophytes, reflecting deep evolutionary tuning to the prevailing light environment, thylakoid organization, genomic constraints, and metabolic economy. Although all PBSs perform the same fundamental task, harvesting light and funneling excitation to the photosystems, the optimal structural solution differs markedly among taxa. Each architecture represents a distinct strategy for maximizing photon capture under a specific combination of ecological and physiological conditions (Adir, 2005; Watanabe & Ikeuchi, 2013). Recent *in situ* cryo‐ET has further demonstrated that PBSs form architecture‐specific supercomplexes with photosystems, revealing that architectural variation is tightly integrated with thylakoid membrane organization (Li et al., 2021; Rast et al., 2019; You et al., 2023; Zhang et al., 2024).

This means that even if two cells possess identical pigment content, differences in PBS architecture alone can lead to substantial variation in excitation energy‐transfer efficiency, photosystem coupling, regulatory flexibility, and ultimately ecological fitness under different light regimes. These functional disparities arise not from pigment quantity *per se* but from the three‐dimensional organization of the light‐harvesting antennae and the spatial arrangement of their chromophores and LPs (Adir, 2005; MacColl, 1998). At the same time, PBS architecture imposes geometric and spatial constraints on how pigments are distributed relative to the photosystems, thereby shaping the effective antenna size and the ratio of light‐harvesting pigments to reaction centers. As a result, architecture not only governs how efficiently pigments function, but also sets and tunes the light‐harvesting antenna size and pigment: photosystem stoichiometry, with direct consequences for photosynthetic performance under varying environmental and metabolic constraints (Grossman et al., 1993).

Architecture also governs PBS‐photosystem docking geometry, influencing the distance and orientation between terminal emitters (ApcE, ApcD, ApcF) and the reaction centers. New evidence shows that specific LPs, such as ApcG, directly modulate PBS–PSII interactions and energy‐transfer efficiency, underscoring the architectural dependence of photosystem coupling (Espinoza‐Corral et al., 2024).

Beyond their photophysical properties, PBS architectures also define the regulatory flexibility of the antenna. Hemidiscoidal PBSs, in particular, possess a broad repertoire of adaptive responses, including CA, OCP‐mediated photoprotective quenching, array formation, and the ability to repurpose peripheral rod assemblies into a dedicated PSI antenna. Different rod conformations may also contribute to environmental responsiveness, although their specific roles remain unknown. Thus, PBS architecture represents an evolutionary response to the availability of light quality and quantity, thylakoid topology, nutrient status, and broader environmental variability, rather than a mere byproduct of pigment biosynthesis.

Taken together, these observations underscore that PBS architecture is a functional trait in its own right, shaping photophysics, membrane interactions, regulatory dynamics, and ecological performance. Even when pigment content is held constant, the three‐dimensional organization of the PBS profoundly influences how effectively an organism captures and uses light. Recognizing architecture as a key adaptive dimension is therefore essential for understanding the diversity, evolution, and ecological distribution of PBSs across cyanobacteria and red algae.

## MARINE CYANOBACTERIAL LIGHT‐HARVESTING

*Prochlorococcus* and *Synechococcus*, known as the marine picocyanobacteria, are abundant unicellular cyanobacteria and major participants in global carbon cycles (Huston & Wolverton, 2009). These α‐cyanobacteria possess a light‐harvesting antenna system that clearly stands apart from that of terrestrial or freshwater cyanobacteria, making it an adaptation worth highlighting (Ting et al., 2002). Because red wavelengths attenuate rapidly with depth, underwater light becomes both dimmer and increasingly blue, and marine cyanobacteria have evolved pigment repertoires that match these spectral conditions. Although they are closely related and often coexist in the same ocean habitat, *Prochlorococcus* and *Synechococcus* possess very different photosynthetic light‐harvesting antennas (Bibby et al., 2001, 2003; Partensky & Garczarek, 2010; Six et al., 2007; Ting et al., 2002). *Prochlorococcus* uses a streamlined chlorophyll *a*_2_/*b*_2_ light‐harvesting complex, whereas many *Synechococcus* clades deploy highly tuned PEs optimized for the blue‐shifted, nutrient‐poor light environment of the open ocean.

*Prochlorococcus* light‐harvesting antenna relies on integral membrane complexes composed of divinyl‐chlorophyll *a* and *b* (Chl *a*_2_ and Chl *b*_2_) bound to *Prochlorococcus*‐specific Pcb (Prochlorophyte chlorophyll‐binding) proteins (Garczarek et al., 2001; La Roche et al., 1996). These proteins do not belong to the LHC superfamily, present in green algae and plants; instead, Pcbs are members of a family that includes the Chl *a*‐binding proteins CP43 and CP47, which form the core antenna associated with the PSII reaction center in all oxygenic photosynthetic organisms. Structural work has revealed low‐resolution Pcb–PSI supercomplexes supporting efficient absorption under extremely low light (Bibby et al., 2001, 2003). In addition, a low‐detail reconstruction shows that Pcb proteins can assemble into a PSII–Pcb supercomplex, forming a dedicated PSII antenna (Bibby et al., 2003; MacGregor‐Chatwin et al., 2019). In addition, *Prochlorococcus* ecotypes possess remanent of the PBS in the form of a single PBP, PE (Hess et al., 1996, 2001). However, the function of this unusual PE in *Prochlorococcus* remains enigmatic, and its presence is particularly intriguing given the highly reduced genomes of *Prochlorococcus* species, in which it is generally assumed that only genes with essential or strongly selected functions are retained (Dufresne et al., 2003; Wiethaus et al., 2010). Recently, it has been shown that the most basal *Prochlorococcus* lineages are adapted to the low‐oxygen, low‐light, and high‐nutrient conditions found in the dimly illuminated waters of anoxic marine zones and have retained PBSs as light‐harvesting antennae (Ulloa et al., 2021), a finding that opens new questions about the evolution and functional diversity of light‐harvesting strategies in this globally important cyanobacterium.

Marine *Synechococcus* species, on the contrary, possess hemidiscoidal PBSs (Kolodny et al., 2021; Six et al., 2007), either in the tricylindrical or pentacylindrical core architecture (Dominguez‐Martin et al., 2022; Zheng et al., 2021). Depending on the ecotype, *Synechococcus* strains incorporate distinct PBP combinations into their PBS rods (Grebert et al., 2022; Six et al., 2007): Pigment type 1 (PT1) contains only PC, absorbing in the red; pigment type 2 (PT2) contains PC and PE‐I (binds either only PEB or both PEB and PUB), absorbing in the green; and pigment type 3 (PT3) contains PC, PE‐I, and the blue‐absorbing PE‐II (binds PUB and PEB). A subset of PT3 strains (PT3d) can further remodel their PBS composition in response to ambient light color through CA, dynamically tuning their absorption properties to shifting spectral environments. Moreover, marine *Synechococcus* lineages exhibit the greatest pigment diversity and the most elaborate rod architectures of any cyanobacteria.

## ENGINEERING AND BIOTECHNOLOGICAL APPLICATIONS OF PHYCOBILISOMES

Minimizing light‐harvesting antenna size, an approach broadly known as the truncated antenna size concept, has emerged as a promising strategy to enhance photosynthetic productivity (Kirst & Melis, 2018; Melis, 2009). Reducing PBS size by deleting the genes that form the rods of the PBS leaving only the core decreases self‐shading, increases light penetration in dense cultures, and can enhance biomass accumulation and product yields under high‐irradiance conditions by enabling more cells to operate photosynthesis efficiently (Kirst et al., 2014). However, work by Lea‐Smith et al. (2014) demonstrated that antenna truncation can also impair growth or reduce photosynthetic efficiency under certain environmental regimes, indicating that the benefits of PBS reduction are context‐dependent and influenced by strain background, culture density, and light quality. Together, these findings highlight both the potential and the complexity of antenna engineering: While PBS truncation can improve bioproduction, its success depends on carefully tuning antenna size to the physiological and environmental constraints of the production system.

Beyond their central role in cyanobacterial photosynthesis, PBS and their constituent pigments have emerged as versatile platforms for biotechnological innovation. The unique structural and photophysical properties of PBPs, particularly their high quantum yields, tunable absorption across the visible spectrum, and remarkable photostability, have positioned them as valuable natural antioxidants with demonstrated potential in biomedical, nutraceutical, and anti‐inflammatory applications (Dagnino‐Leone et al., 2022; Fernández‐Rojas et al., 2026; Patel et al., 2005; Romay et al., 1998). At the same time, the deepening mechanistic understanding of PBS assembly, energy‐transfer pathways, and spectral tuning functions as a blueprint for bioinspired and hybrid light‐harvesting systems.

An emerging application of PBS proteins is their use as fusion partners for the stable over‐expression of heterologous proteins in cyanobacteria (Formighieri & Melis, 2015; Melis et al., 2024). Foreign proteins can be expressed at remarkably high levels when fused to the abundant β‐subunit of PC (CpcB) (Majhi & Melis, 2025). The resulting fusion polypeptides assemble into functional PC discs rather than remaining free in the cytosol, enabling stable accumulation of the recombinant protein to 10–15% of total cellular protein (Hidalgo Martinez et al., 2022). Similar constructs using CpcA or the rod‐linker CpcG1 as fusion partners also form functional antenna discs and support high‐level expression (Hidalgo Martinez et al., 2022), establishing PBS components as robust scaffolds for photosynthetic bioproduct generation.

Furthermore, PBPs such as PC and PE are increasingly explored as natural food colorants, with demonstrated applications in dairy beverages and milk‐based products, as well as growing industrial interest driven by their vivid coloration and antioxidant properties (Carabantes & Dufossé, 2026; Galetovic et al., 2020; Minic et al., 2024).

By uncovering the full diversity and mechanistic sophistication of PBSs, future research will not only strengthen today's applications but also catalyze unforeseen uses of these modular light‐harvesting systems in biotechnology.

## CONFLICT OF INTEREST

The authors have no conflict of interest.

## Data Availability

Data sharing not applicable to this article as no datasets were generated or analyzed during the current study.
